# Trunk muscle activity in healthy subjects during bridging stabilization exercises

**DOI:** 10.1186/1471-2474-7-75

**Published:** 2006-09-20

**Authors:** Veerle K Stevens, Katie G Bouche, Nele N Mahieu, Pascal L Coorevits, Guy G Vanderstraeten, Lieven A Danneels

**Affiliations:** 1 Department of Rehabilitation Sciences and Physiotherapy, Faculty of Medicine and Health Sciences, Ghent University, Belgium

## Abstract

**Background:**

Trunk bridging exercises are often used as therapeutic exercises for lumbopelvic stabilization. These exercises focus on the retraining of muscle coordination patterns in which optimal ratios between local segmental stabilizing and global torque producing muscle activity are assumed to be essential. However, a description of such ratios is lacking. The purpose of this study was to investigate both relative (as a percentage of maximal voluntary isometric contraction) muscle activity levels and ratios of local to global muscle activity, during bridging stabilization exercises.

**Methods:**

Thirty healthy university students (15 men, 15 women) with a mean age of 19.6 year volunteered to perform 3 bridging exercises (single bridging, ball bridge and unilateral bridging). The surface electromyographic activity of different trunk muscles was evaluated on both sides.

**Results:**

During all bridging exercises, the ratio of the internal oblique to the rectus abdominis was very high due to minimal relative activity of the rectus abdominis. In general, the ratio of the internal/external abdominal oblique activity was about 1. However, during the unilateral bridging exercise, the ipsilateral internal/external abdominal oblique activity ratio was 2.79 as a consequence of the significant higher relative activity of the internal oblique compared to the external oblique. The relative muscle activity and the ratios of the back muscles demonstrated similar activity levels for all back muscles, resulting in ratios about 1.

**Conclusion:**

Both the minimal relative activity of the rectus abdominis and the high internal oblique to the rectus abdominis activity ratio reported in the present study are in accordance with results of other trunk stabilization exercises. The relative muscle activity and the ratio of the abdominal obliques seem to alter depending on the task and the presumable need for stability. The findings concerning the relative muscle activity and the ratios of the back muscles support the assumption that during these bridging exercises, all back muscles contribute in a similar way to control spine positions and movements in a healthy population.

## Background

Stability and movement are determined by the coordination of all the muscles that surround the lumbar spine [1-3]. A strategy of trunk stiffening on one hand and creating optimal movement on the other hand is assumed to be essential [1]. Within this context, stabilization exercises are often used in clinical practice today. The main goal of stabilization exercises is to protect spinal joint structures from further repetitive microtrauma, recurrent pain and degenerative change [4]. Long term results of various studies seem to indicate that specific lumbar stabilizing therapy as a single therapy or combined with other treatments can reduce the intensity of the pain and disability in low back pain (LBP) [5-8] and pelvic girdle pain patients [9,10] and prevent recurrent pain episodes [11-13].

Debate exists on the anatomical classification of muscles in local and global muscles related to specific functions, respectively segmental stabilizing (local) and torque producing and providing general trunk stability (global), as proposed by Bergmark [14]. Some mentioned that this classification is incorrect since no single muscle is superior at enhancing stability [2,15,16]. In line with this, assessment of some "stabilization" exercises revealed that no individual muscle could create an unstable situation when artificially reduced in activation [15]. During stabilization training, Marshall & Murphy [17] aimed at minimizing rectus abdominis (RA) activity in comparison with all other muscles of the lumbopelvic region. In contrast, other researchers assumed that, once an optimal local activation has been achieved, the interplay between local and global muscles is thought to be necessary [18,19]. To meet the different opinions, analysis of both so-called local and global muscles was considered necessary. More than evaluation of differences between relative muscle activity levels of local and global muscles, ratios of relative muscle activity were thought to provide insight into the contribution of both muscle systems in relation to each other.

In the past, ratios of local to global muscle activity were only analysed in specific isolated local muscle contraction tasks (abdominal drawing in manoeuvre) [20] and general movement and isometric contraction activities (flexion, extension and lateral flexion from a semiseated position in an apparatus) [21]. Recently, the ratio of the relative internal abdominal oblique (IO) to rectus abdominis (RA) activity was reported in a small population performing core stability exercises on and off a swiss ball [17]. However, the contribution of both local and global muscles calculated as a ratio was currently not analysed in briding exercises.

The present study focused on 3 different bridging exercises often used early in a lumbar stabilization training program. The supine posture with the knees and hips bent used during bridging exercises, is to most LBP patients a comfortable, pain-free posture. From this position limited movements, such as lifting the pelvis, can be started. In order to create more functional tasks, limb movements can be added. By combining pelvis and leg movements as used in exercise 3 in the present study, it is hypothesized that more global muscle activity will be required to perform those more demanding tasks [15].

Exercise 2 in the present study was a ball bridge stabilization exercise. To amplify the training effects of a bridging exercise and specifically challenge stability mechanisms, labile surfaces such as gymnastic balls used to be advised [22,23]. However, recent research evaluating bridging [24,25], other stabilization exercises [17,26] and trunk extension exercises [27] could not support that the use of an exercise ball can create a greater challenge for the musculoskeletal system or a training advantage in a healthy population.

The purpose of this study was to investigate the relative (% of maximal voluntary isometric contraction) muscle activity and ratios of local to global muscle activity during single bridging stabilization exercises, ball bridging exercises and bridging exercises with leg movements.

## Methods

### Subjects

Thirty healthy university students (15 men and 15 women) voluntary participated to the study. Subjects had no history of neurological, respiratory or musculoskeletal back or lower limb pathology. All subjects had an 'average' activity level, as determined by the Dutch version of the habitual physical activity questionnaire [28]. They had a mean age of 19.6 (range:19–23) year, a mean height of 176.6 (range:157–194) cm and a weight of 66.9 (range:42–84) kg. All subjects signed an informed consent. The subjects had no experience with stabilization principles. The protocol was approved by the Ethics Committee of the Ghent University Hospital.

### Electromyography (EMG) preparation

Prior to the experimental phase, each subject was prepared for EMG recording as follows. The skin was prepared by shaving excess hair and rubbing the skin with alcohol to reduce impedance (typically ≤ 10 kOhm). Disposable Ag/AgCl surface electrodes (Bleu Sensor, Medicotest GmbH, Germany) were attached parallel to the muscle fibre orientation, bilaterally over the following so-called local trunk muscles: the inferior fibres of the IO (midway between the anterior iliac spine and symphysis pubis, above the inguinal ligament)[29,30], the lumbar multifidus (MF) (lateral to the midline of the body, above and below a line connecting both posterior superior iliac spines)[31,32] and the lumbar part of the iliocostalis lumborum (ICLL) (lateral to the vertical line through the posterior superior iliac spine, above the iliac crest)[32]. The inferior fibres of the IO were considered to represent local muscle activity [4,14] because it was shown that on the site medial and inferior to the anterior superior iliac spine, the fibres of the transversus abdominus and the IO are blended, so a distinction between the muscle signals cannot be made at this location [33]. Concerning the back muscles, the MF and ICLL were so-called local muscles because of their direct attachments to the vertebrae [4,14]. Because the RA, the external abdominal oblique (EO) and the thoracic part of the iliocostalis lumborum (ICLT) transfer the load directly between the thoracic cage and the pelvis, some call them global trunk muscles [4,14]. The electrode placement of those global trunk muscles was as follows: the EO (15 cm lateral to the umbilicus)[22,29,30,34,35], the RA (3 cm lateral to the umbilicus)[30,34,36,37] and the ICLT (above and below the L1 level, midway between the midline and the lateral aspect of the body)[30,32]. The maximum interelectrode spacing between the recording electrodes was 2.5 cm as recommended by Ng et al. [38], and each electrode had an approximately 1.0 cm^2 ^pick-up area.

### Maximal voluntary isometric contraction (MVIC) assessment

The MVICs of the muscles were measured in three trials before the experimental tasks. These exercises were performed to provide a basis for EMG signal amplitude normalization [30,34,35,39-43]. Normalization of EMG corresponding maximal EMG amplitude allows interindividual comparison to the individual maximum [44]. Failure to normalize EMG data before quantitative analysis introduces confounding variables not related to muscle function (for example skin impedance, electrode orientation and amount of subcutaneous tissue) [44]. Five different isometric exercises against manual resistance were executed. Verbal encouragement was given to ensure maximal effort. The maximal activation of the abdominal obliques (IO and EO) was obtained by a combined flexion-rotation exertion from a supported, straight-knee sitting position, with the hands placed behind the head and the trunk held in a 45° angle. Manual resistance was applied to the contralateral shoulder [30,39]. From the same sitting position the subject was asked to perform a trunk flexion against bilateral manual resistance applied to both shoulders, for the generation of the maximal isometric activity of the RA [39,43]. Concerning the MVICs of the MF [29,30,34,42,43] and the lumbar and thoracic part of the iliocostalis lumborum (ICLL and ICLT)[29,30,43] manual resistance was applied to the posterior aspect of the scapula while the subject lay in the prone position, with the legs strapped to the table to prevent them from moving. The subject was asked to perform a trunk extension.

### Procedures and instrumentation

**Figure 1:**
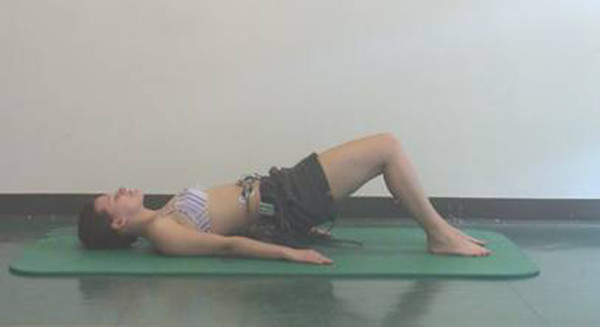
Single bridging exercise (exercise 1).

**Figure 2:**
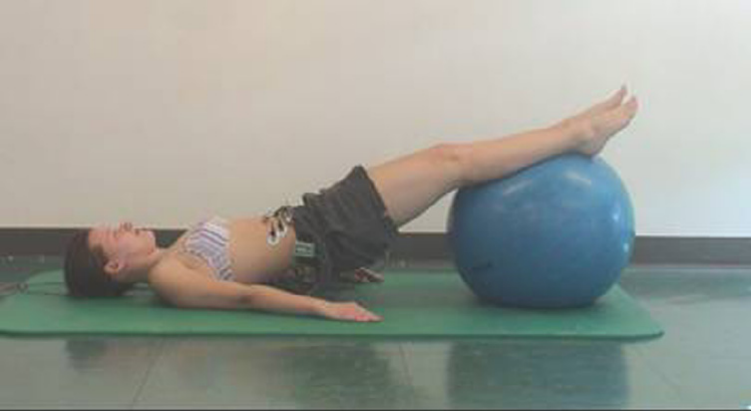
Ball bridge exercise (exercise 2).

**Figure 3:**
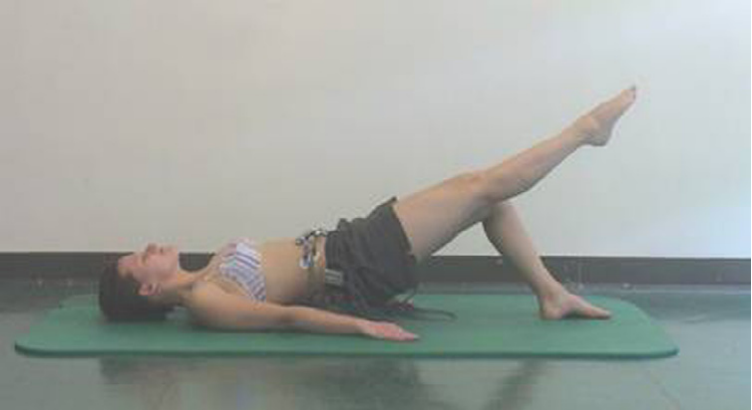
Unilateral bridging (exercise 3).

The subjects performed 3 experimental exercises, often used in clinical practice to train the stability of the lower back. These exercises were executed in supine position, knees bent (60° flexion) and feet on the floor. Exercise 1 was a single bridging exercise (figure 1), exercise 2 a ball bridge exercise (figure 2) and exercise 3 a bridging exercise with extension of the left or right leg (unilateral bridging exercise) (figure 3). Exercises 1 and 2 can be called symmetric exercises and exercise 3 is an asymmetric exercise. After a detailed explanation of each exercise, followed by a guided trial, the exercises were recorded. The subjects lifted their pelvis until an angle of zero degrees hipflexion was reached. At the beginning of each exercise a neutral lumbar spine position was determined by the examiner (anterior and posterior iliac spines in line)[3] and the subject was encouraged to hold this position during the course of the total exercise. To standardize the position of the subject and the equipment, markers were placed on the floor. The exercises were executed in a random sequence. The dynamic phases, lifting and lowering of the pelvis and the extremities, lasted two seconds. The bridged positions in exercises 1 and 2 and the leg extension in exercise 3 were hold for five seconds. The pace of 60 beats/min was set by a metronome. Three trials for every exercise were performed. A pause of at least 15 seconds was allowed between the trials.

The raw surface EMG signals were bandpass-filtered between 10 and 500 Hz and amplified using a differential amplifier (MyoSystem 1400, Noraxon Inc, Scottsdale, AZ). The overall gain was 1000 and the common mode rate rejection ratio was 115 dB. The signals were analogue/digitally (A/D) (12-bit resolution) converted at 1000 Hz and stored in a personal computer.

### Data analysis

The stored data were full-wave rectified and smoothed with a root mean square (RMS) with a window of 150 milliseconds. For each of the muscles and for each testing session, the RMS was calculated for the 3 repetitions of the different exercises. The mean RMS of the three MVIC trials for every muscle was used to provide a basis for EMG signal amplitude normalization of the data of the experimental exercises. The static phases of the exercises were analysed, using an interval of 4700 ms after the defined starting point of the holding position. Noraxon MyoResearch software 2.10 was used.

Not only the relative muscle activity of different trunk muscles, but also ratios of the relative local abdominal muscle activity to the global abdominal muscle activity (IO/RA and IO/EO) were calculated. In a similar way, ratios of the relative back muscle activity (MF/ICLT and ICLL/ICLT) were determined.

### Statistical analysis

Statistical analysis was performed using SPSS 12.0 software package (SPSS Inc., Chicago, IL) for Windows. The level for statistical significance was set at α = 0.05. As there was no significant difference between the muscle activity of the left and right muscles during exercise 1 and 2, the mean activity levels were used. There were also no significant differences between the muscle activity at the left side when performing a left leg extension and the muscle activity at the right side when performing a right leg extension (exercise 3). Therefore the mean value was used for further analysis and called ipsilateral muscle activity. In accordance with the same findings on the other side, the new term contralateral muscle activity was introduced. Concerning both the MVICs and the experimental exercises, an analysis of variance for repeated measures was applied to evaluate the effects of the factor muscle during every single exercise, separately for the abdominal and the back muscles. Since the factor abdominal muscle was significant (p < 0.001) during all exercises and the factor back muscle was significant (p = 0.02) during the ball bridge exercise, post-hoc least significance difference tests (LSD), adjusted by a Bonferroni test to protect against type I errors, were used to analyze the significant differences between the individual muscles in each exercise. Descriptive statistics showed the relative abdominal and back muscle activity ratios.

## Results

The mean EMG amplitudes of the different abdominal and back muscles during the 3 bridging exercises are presented in Table 1. Since particularly the contribution of the local muscle activity compared to global muscle activity is concerned, the analysis of the abdominal muscle activity is performed separately from the analysis of the back muscle activity.

Concerning the abdominal muscles, during all exercises, the relative activity of the RA was significantly lower than the relative activity of the obliques (p < 0.001). During the single bridging exercise 1, the muscle activity of the obliques did not differ significantly (p = 1.00). In contrast, during the ball bridge exercise 2, the EO showed significantly higher activity levels than the IO (p = 0.003). During exercise 3, the contralateral EO activity was also significantly higher than the contralateral IO activity (p = 0.007), but the ipsilateral EO activity was significantly lower than the ipsilateral IO activity (p < 0.001).

Regarding the back muscles, except for the ICLL, which showed significant higher activity than the ICLT during the ball bridge exercise 2 (p = 0.01), the activity levels did not differ significantly (p ≥ 0.26).

**Figure 4:**
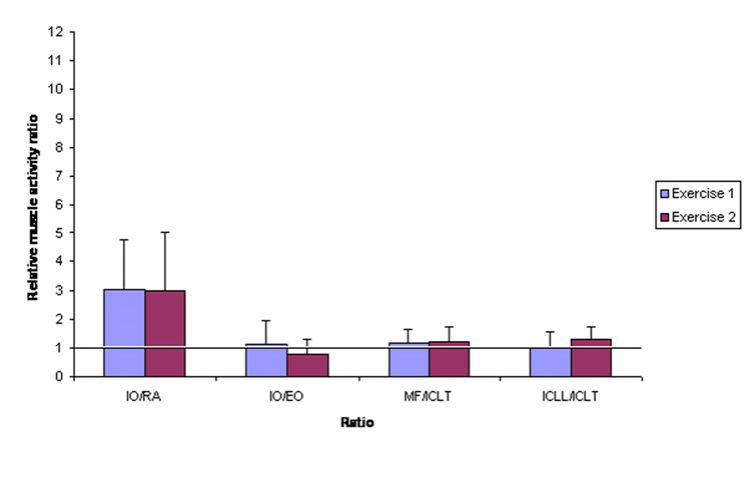
**Mean ratios and SD of relative local muscle activity to relative global trunk muscle activity during the single bridging (exercise 1) and ball bridge exercise (exercise 2)**. IO = internal oblique; MF = lumbar multifidus; ICLL = iliocostalis lumborum pars lumborum; RA = rectus abdominis; EO = external oblique; ICLT = iliocostalis lumborum pars thoracis.

**Figure 5:**
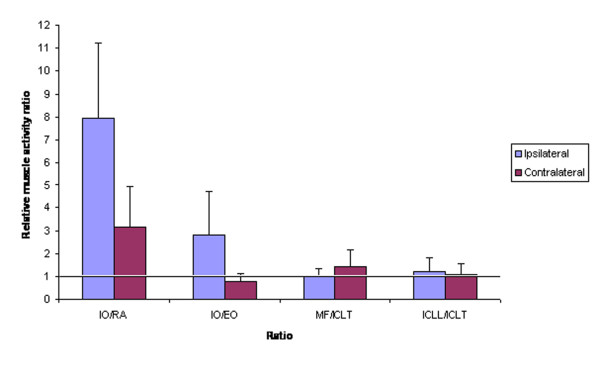
**Mean ratios and SD of relative local muscle activity to relative global trunk muscle activity during the unilateral bridging exercise (exercise 3)**. IO = internal oblique; MF = lumbar multifidus; ICLL = iliocostalis lumborum pars lumborum; RA = rectus abdominis; EO = external oblique; ICLT = iliocostalis lumborum pars thoracis.

To emphasize the relation between so-called local segmental stabilizing muscles and global torque producing muscles, the relative activity was expressed as ratios. The mean ratios are presented in figures 4 and 5.

In general, the ratio of the local to the global muscle activity was about 1.

The IO/RA ratio was much higher than 1 during all exercises (3.00 in exercise 1 and 2.96 in exercise 2). During exercise 3, the ipsilateral IO/RA was 7.95 and the contralateral IO/RA was 3.16. The ipsilateral IO/EO was higher during exercise 3 (2.79).

The MVICs were used to normalize the EMG values obtained during the experimental exercises. The mean EMG amplitudes and standard deviations (SD) are presented in Table 2. The MVICs of the back muscles did not differ significantly (p ≥ 0.60). Except for the significant higher MVIC of the RA in comparison with the MVIC of the IO and EO (p ≤ 0.01), the abdominal muscles did not show significant different amplitudes (p = 1.00).

## Discussion

The purpose of the current study was to evaluate the muscle activity during commonly used bridging stabilization exercises. The investigated exercises are supposed to be beneficial to stabilize the lumbar spine region. When describing exercise therapy, it is important to understand the muscle activity in healthy conditions. In the current study the muscle activity is expressed as relative (as a percentage of MVIC) EMG as well as ratios of relative activity. The description of differences in activation patterns of so-called local and global muscles can be made more sensitive by calculating ratios than using isolated relative muscle activity levels [20]. Some researchers and clinicians assume that optimal stabilization of the lower back during basic stabilization exercises may be created by a good activation of the local muscles [4,19,45-47]. In this respect, the way the local muscle activity is related to the global muscle activity can be assumed more important than the relative muscle activity levels of the muscles separately. For interpretation purposes, the results of both the relative EMG activity and the ratios of relative activity are integrated.

Concerning the *abdominal muscles*, during all bridging exercises, the relative activity of the RA was significantly lower than the relative muscle activity of the obliques. One of the consequences of this low relative RA activity was that during all exercises, the IO/RA ratio demonstrated the highest values compared to the other abdominal and back muscle activity ratios. However, these findings need to be interpreted with caution, since normalization of the EMG data of the experimental exercises occurred using MVICs and the RA MVIC amplitude was significantly higher in comparison with the MVIC amplitudes of the abdominal obliques. Nevertheless, the consistent low-level activity of the RA was in accordance with the findings of similar research [36,48] and research of related exercises [39]. These studies also used MVIC normalization procedures, but did not report the MVIC values nor analysis performed on these data.

The small relative muscle activity levels reported in the present and the latter studies were not necessarily related to a nonstabilizing capacity of this muscle. Only small activity levels seem to be necessary to ensure sufficient stability in a neutral spine posture in non-weightbearing positions [40]. Generally for most tasks of daily living very modest levels of abdominal wall co-contraction are sufficient [2]. Cholewicki et al. [40] highlighted the importance of motor control to coordinate muscle recruitment between so-called global and local muscles during functional activities to ensure mechanical stability is maintained. Under such conditions they suggested that intersegmental muscle activity as low as 1 to 3 % MVC may be sufficient to ensure dynamic stability [40]. Furthermore, biomechanical modelling is needed to draw conclusions about stability contributions as stability is also proportional to the square of the muscle's moment arm.

The objective for the use of the ratio of IO/RA activity in the present study, was to enhance the understanding of the co-activation of both local and global muscles during this kind of stabilization exercises. Other researchers stated that analysis of the IO/RA ratio is important to verify if the activity of the RA is minimal in comparison with all other muscles of the lumbopelvic region to fulfil the requirement for a good stabilization exercise [17]. In our opinion, respecting adequate activation levels depending on the demands during different tasks is essential, rather than aiming at minimal activity of certain muscles. Ratios assist in providing further insight in the co-operation of the different muscles during various tasks.

During the ball bridge exercise, the EO showed significantly higher relative EMG than the IO. Consequently, the IO/EO ratio was low (< 1) during this exercise. In accordance with these results, McGill [1] assumed that the EO may have a greater potential in stabilizing the trunk than the local abdominal muscles. Vera-Garcia et al. [29] found that when performing curl-ups on a gymnastic ball, there was much more co-contraction of the EO muscle with the RA muscle when compared to other tasks because of the greatest possibility of rolling laterally off the ball. In order to enhance this stability, it appears that the motor control system selects to increase EO activity more than the other abdominal muscles. However, recent research evaluating bridging exercises showed no significant differences in relative EO and RA activity between performance on firm or ball surfaces [24,25]. Debate exists on increased [24] or unchanged IO activity [25] during ball bridge excises. However, the ball bridge exercises described in the latter studies were performed with the feet flat on the ball, in contrast to the calf position on the ball in the present study. Although only the calfs were positioned on the ball, the global torque producing EO might be activated more than the local segmental stabilizing IO to prevent the limbs from rolling of the ball and jepardizing the trunk stability. Analysis of the relative EMG activity levels showed a greater increase in EO activity compared to IO activity between the single bridging and the ball bridge exercise. This could explain the small ratio of the IO to EO during the ball bridge exercise in the current study.

During the unilateral bridging exercise, the ipsilateral IO showed significantly higher EMG than the ipsilateral EO and the contralateral IO demonstrated significantly lower activity than the contralateral EO. Consequently, the contralateral IO/EO ratio was low (< 1) and the ipsilateral IO/EO ratio was higher than 2 during this exercise 3. Kavcic et al. [15] reported that during single and unilateral bridging exercises the IO and EO seem to demonstrate consistently a large impact on induced increasing and decreasing stability. Both so-called local and global oblique muscles seem to work together and may have an important role in controlling the neutral spine position during this exercise. When the contralateral leg is raised, a rotational moment about the spine is expected to occur. The ipsilateral IO can cause an ipsilateral rotational moment about the spine and the ipsilateral EO can create a moment in the opposite direction to counter the spine moment. To stop the spine from twisting, appropriate muscle activity may generate stability. The ratio of the IO to EO activity seems to depend on the task and the presumable need for stability.

Regarding the *back muscles*, in general, the relative muscle activity levels of the local and global muscle system were not significantly different. All ratios of relative back muscle activity were about 1. Van Dieën et al. [21] reported ratios of the lumbar to the thoracic erector spinae (ES) muscles, representing local to global muscle activity, varying from 0.5 to 0.9 in healthy subjects during global exercises in a semi-seated posture. Maximal isometric extension exercises seemed to create a lumbar ES/thoracic ES ratio of 1.1 [21]. This demonstrates that during different tasks and exercises, all back muscles contribute in a similar way to control spine positions and movements. These findings support the statement that no single muscle seems superior to another and that all muscles act together in the same way to create a stable position of the spine during this kind of exercises [2,15,16].

Only during the ball bridge exercise, the ICLL showed significantly higher activity than the ICLT. In the past, application of unstable surfaces such as a ball was supposed to increase muscle activity [29]. Since the ICLL is located closer to the centre of rotation than the ICLT, the increasing effect might be higher. However, recent research comparing exercise surfaces in stabilization and trunk extension exercises, demonstrated that the addition of a ball did not influence [17,24-27] or even decreased back muscle activity [27]. In the present study, the ratio ICLL/ICLT remained about 1.

Though the data on the ratios of local to global relative muscle activity were normally distributed, relatively large SDs were noticed concerning the abdominal muscles. These findings represent abdominal ratios spread apart and a relatively flat bell curve, indicating that relatively more subjects showed ratios towards one extreme or the other. Since the mean relative abdominal muscle activity ratios discussed in the present study were supposed to be confined by the spread values, interpretation might be influenced.

LBP patients might demonstrate different recruitment patterns, for instance higher or lower muscle activity due to pain adaptation or spasm caused by pain [49]. Within this context, the current preliminary data may provide a foundation to help determining exercise treatment approaches intended to recruit specific muscle sites.

## Conclusion

To enhance the understanding of the trunk muscle recruitment patterns during stabilization exercises often used in clinical practice, relative EMG activity as well as ratios of muscle activity of both local and global muscles seem important. The present study shows that the abdominal muscle activity ratios IO/RA and IO/EO demonstrate a different pattern. During all exercises, the IO/RA ratio is very high due to minimal RA activity. The relative muscle activity and the ratio of the abdominal obliques seem to alter depending on the task and the presumable need for stability. The findings concerning the relative muscle activity and the ratios of the back muscles support the assumption that during these bridging exercises, all back muscles contribute in a similar way to control spine positions and movements in a healthy population.

## Competing interests

The author(s) declare that they have no competing interests.

## Authors' contributions

VKS participated in the study design, in collecting the data, the statistical analyses, and drafting of the manuscript. KGB and NNM participated in the design of the study. PLC advised and assisted in the statistical analyses. GGV coordinated the study. LAD participated in the study design and in the progress and drafting of the manuscript. All authors read and approved the final manuscript.

## Pre-publication history

The pre-publication history for this paper can be accessed here:



## Figures and Tables

**Table 1:** Mean relative muscle activity (% of MVIC) and standard deviation (SD) of the different trunk muscles during bridging exercises.

	**IO**		**RA**		**EO**		**MF**		**ICLL**		**ICLT**	
	**Mean**	**(SD)**	**Mean**	**(SD)**	**Mean**	**(SD)**	**Mean**	**(SD)**	**Mean**	**(SD)**	**Mean**	**(SD)**
	
**Exercise 1**	5.58	(5.00)	1.91	(1.27)	5.98	(4.36)	22.64	(7.51)	20.32	(8.56)	20.59	(5.51)
**Exercise 2**	6.58	(4.80)	2.76	(2.35)	10.32	(7.99)	23.99	(7.15)	27.17	(10.20)	22.24	(6.96)
**Exercise 3**												
*Ipsilateral*	29.80	(9.97)	4.72	(3.45)	16.34	(12.09)	23.54	(6.33)	28.45	(11.50)	25.84	(7.84)
*Contralateral*	10.11	(6.95)	3.55	(2.18)	14.93	(9.34)	24.58	(8.80)	20.44	(9.24)	20.60	(8.22)

IO = internal oblique; MF = lumbar multifidus; ICLL = iliocostalis lumborum pars lumborum; RA = rectus abdominis; EO = external oblique; ICLT = iliocostalis lumborum pars thoracis.

**Table 2:** Mean EMG activity (Mean) and standard deviation (SD) of the maximal voluntary isometric contractions.

	**Mean**	**(SD)**
	
**IO**	184.78	(82.94)
**RA**	275.96	(132.06)
**EO**	191.50	(136.74)
**MF**	264.23	(114.93)
**ICLL**	254.17	(147.80)
**ICLT**	240.63	(106.78)

IO = internal oblique; MF = lumbar multifidus; ICLL = iliocostalis lumborum pars lumborum; RA = rectus abdominis; EO = external oblique; ICLT = iliocostalis lumborum pars thoracis.
